# Microglia and Spinal Cord Synaptic Plasticity in Persistent Pain

**DOI:** 10.1155/2013/753656

**Published:** 2013-08-18

**Authors:** Sarah Taves, Temugin Berta, Gang Chen, Ru-Rong Ji

**Affiliations:** Pain Signaling and Plasticity Laboratory, Departments of Anesthesiology and Neurobiology, Duke University Medical Center, Durham, NC 27710, USA

## Abstract

Microglia are regarded as macrophages in the central nervous system (CNS) and play an important role in neuroinflammation in the CNS. Microglial activation has been strongly implicated in neurodegeneration in the brain. Increasing evidence also suggests an important role of spinal cord microglia in the genesis of persistent pain, by releasing the proinflammatory cytokines tumor necrosis factor-alpha (TNF**α**), Interleukine-1beta (IL-1**β**), and brain derived neurotrophic factor (BDNF). In this review, we discuss the recent findings illustrating the importance of microglial mediators in regulating synaptic plasticity of the excitatory and inhibitory pain circuits in the spinal cord, leading to enhanced pain states. Insights into microglial-neuronal interactions in the spinal cord dorsal horn will not only further our understanding of neural plasticity but may also lead to novel therapeutics for chronic pain management.

## 1. Microglia-Synapse Interactions in Healthy CNS

Microglia are derived from myeloid precursor cells in the periphery and penetrate the central nervous system (CNS) during embryogenesis [1]. They account for approximately 10–20% of all cells in the CNS, however their distribution varies from one region to another [2, 3]. Microglial density is particularly high in the hippocampus, basal ganglia, substantia nigra, and spinal cord [2, 4]. Microglia are regarded as the resident macrophages in the CNS and, similar to peripheral macrophages, they display different morphology depending upon their physiological states. In the resting physiological state, microglial cells are ramified with slender, radially projecting processes with similar thickness, length, and ramification, whereas in pathological states, microglia can be activated presenting an amoeboid morphology characterized by large soma, short/thick, and radially projecting processes with few ramifications [4–6]. Although most studies have focused on the role of activated microglia and synaptic transmission, both resting and activated microglia dynamically interact with synapses shaping their connectivity and function [7].

Microglial processes constantly and dynamically survey their environment and interact with nearby synapses [8, 9]. In mature CNS, it has been observed that microglial processes interact with axon terminals and dendritic spines in a transient manner, for an average of five minutes and at a rate of approximately one microglial contact per hour [10]. Notably, microglia processes are driven by neuronal activity and can simultaneously interact with both presynaptic and postsynaptic elements. Reducing neural activity by inhibiting sensory inputs or lowering body temperature results in retraction of microglial processes and decreases the frequency of contacts between microglial processes and synapses [10]. It is well known that astrocyte processes envelop synapses and actively modulate physiological synaptic transmission; however, whether and how microglia directly influence physiological synaptic transmission is still unclear. 

Several studies demonstrated that microglial processes can engulf synapses and participate to their phagocytic elimination in an experience-dependent manner in the mature healthy brain [11–13]. Interestingly, a progressive accumulation of microglial phagocytic-like structures was observed in both mouse visual and auditory cortices by age-related loss of vision and hearing, respectively [14]. This suggests that microglia shape neuronal circuits not only during postnatal development but aslo along the lifespan. Together, these observations suggest that periodic interactions between microglia and synapses exist in the absence of pathological insult. These interactions may be compromised following nervous system injury or disease. The physiological role of microglia in spinal cord circuitry development and pain transmission remains to be investigated. 

## 2. Nociceptive Pain and Persistent Pain

Our bodies play host to a wide variety of sensory information that is detected every moment by the peripheral nervous system. Primary sensory neurons that are responsible for the detection and transduction of painful stimuli (e.g., cold, heat, mechanical, and chemical), which are somatosensory stimuli that cause potential danger to the organism, are called nociceptors [15]. Nociception is of vital importance for survival; thus, it has become a highly regulated pathway within the nervous system of humans. Nociceptive input elicits pain as well as emotional, neuroendocrine, and autonomic responses. Persistent nociceptive input in this system after intense noxious stimulation or tissue injury results in activity-dependent plasticity or a progressive increase in the response of the system to repeated stimuli [16]. As a result of the neural plasticity, a normally innocuous low-threshold stimulation such as light touch could trigger a painful response (mechanical allodynia). Pathological pain or chronic pain results from neural plasticity both in the peripheral nervous system (i.e., peripheral sensitization) and CNS (central sensitization) [17]. The circuitry in the spinal cord dorsal horn connects incoming primary afferents to outgoing projection neurons that ascend to the brain. The projection neurons in the superficial dorsal horn (lamina I) also receive input from interneurons in the lamina II [18]. Importantly, spinal cord dorsal horn neurons undergo marked plastic changes in the pathological conditions, leading to hyperactivity of the projections neurons. Thus, dorsal horn is not only a critical relay center in nociceptive transmission [19] but also an important player in the development and maintenance of central sensitization [16, 17]. Several animal models are used for the study of neuropathic pain. In chronic constriction injury (CCI), the sciatic nerve is constricted by several loose ligatures. In spared nerve injury (SNI), the tibial and common peroneal divisions of the sciatic nerve are ligated and cut while sparing the sural division. In spinal nerve ligation (SNL), a spinal nerve, usually L5, is ligated where it exits the spinal column. Each of these models produces robust mechanical allodynia for weeks following injury; however, the underlying mechanisms may vary. Here we will focus on the contribution of spinal cord microglia to central sensitization in nerve injury-induced neuropathic pain and tissue injury-induced inflammatory pain states.

## 3. Spinal Cord Microglial Activation in the Context of Persistent Pain

**Figure 1:**
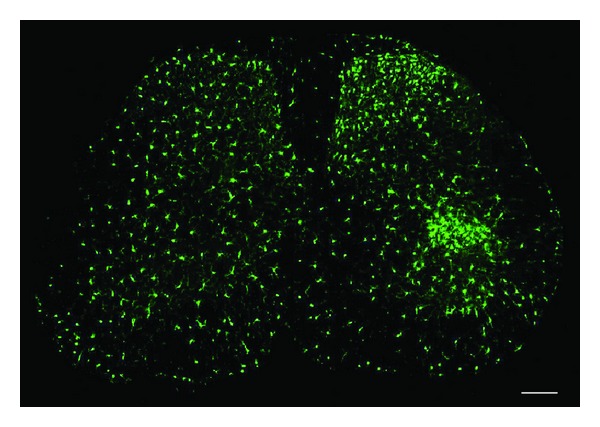
Nerve injury induces marked microglial reaction in the ipsilateral lumbar spinal cord 7 days after chronic constriction injury (CCI). Microglia as demonstrated by CX3CR1 expression in CX3CR1-GFP mice. The contralateral side (left) shows the typical resting microglial morphology, and the ipsilateral side (right) illustrates the enlarged and amoeboid morphological features of activated microglia cell bodies. Also note that on the ipsilateral side the number and density of microglia in the medial superficial dorsal horn and lateral ventral horn, where injured primary afferent axons terminate, are significantly increased. Scale, 100 *μ*m.

Under pathological conditions, especially nerve injury conditions, microglia undergo “microgliosis”, a complex set of changes that allow the cell to respond rapidly and perform a broad range of functions such as shielding injury sites, phagocytosing cellular debris, and releasing inflammatory signals to initiate and/or propagate the immune response. Traditionally, microgliosis has been determined by a change in morphology from ramified to amoeboid [20]. Several pain models of persistent neuropathic pain including CCI, SNI, SNL, or spinal cord injury as well as chronic morphine-induced hyperalgesia and tolerance are associated with the development of microgliosis in the dorsal horn of the spinal cord [21–23].  Figure 1 shows microglial reaction in the dorsal horn of the lumbar spinal cord seven days following CCI of the sciatic nerve in CX3CR1-GFP mice. CX3CR1 promoter activity is restricted to microglia [24]. GFP expression revealed evenly distributed resident microglia in the contralateral side of the spinal cord that exhibited a quiescent or resting type morphology (left, Figure 1). However on the side ipsilateral to injury (right, Figure 1), microglia of the dorsal horn and ventral horn showed enlarged and amoeboid morphology, indicating their activation. Nerve injury also induces marked increases in the density and number of microglia, due to proliferation and possible migration (Figure 1) [25]. Of interest, nerve injury-induced microgliosis is very mild in young animals (2-3 weeks old) [26]. It should be noted, however, that there are also a number of persistent pain conditions that are not associated with spinal cord microgliosis, such as adjuvant-induced inflammatory pain and chemotherapy-induced neuropathic pain [27, 28].

Mitogen-activated kinase (MAPK) pathways are important for intracellular signal transduction and play critical roles in neuronal plasticity and inflammatory responses. The MAPK family consists of three separate signaling pathways: extracellular signal-regulated kinases (ERK), p38, and c-Jun N-terminal kinase (JNK). MAPK activation is correlated with most if not all persistent pain conditions [29, 30]. Several neuropathic pain models, including spinal nerve injury and spared nerve injury, exhibit a robust increase in p38 activation (phosphorylation) in microglia of the dorsal horn beginning at twelve hours, peaking at three days, and slowly declining over several weeks [29, 31–33]. Intrathecal administration of p38 inhibitors has been shown to attenuate neuropathic pain [29, 33]. Minocycline, a broad-spectrum antimicrobial tetracycline compound that inhibits microglial activation, also decreases pain behavior following nerve injury [34, 35], possibly by inhibiting p38 activation [33]. However, minocycline is not able to reverse existing pain states [36]. Interestingly, intrathecal administration of minocycline in models of inflammatory pain, where morphological activation of microglia is not evident, also prevents the development of mechanical sensitization, by inhibiting spinal cord microglial p38 activation [37, 38]. Thus the release of inflammatory mediators from microglia may also occur without morphological alteration. The morphological changes associated with microgliosis may be mediated by the activation of ERK/MAPK [30], and nerve injury was shown to induce ERK activation in spinal microglia in the early phase [39]. It seems there may be multiple activation states whereby microglia do change the manner with which they participate in neural plastic changes, but do not reach a morphologically activated phenotype.

**Figure 2:**
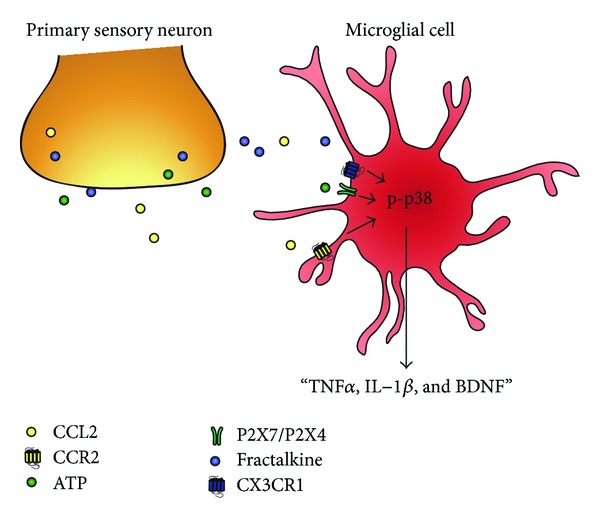
Schematic of primary afferent releasing factors that are responsible for microglial activation. Microglial cells are activated by factors released from hyperexcitable primary afferents such as CCL2 (MCP-1), ATP, and CX3CL1 (fractalkine). Respective activation of CCR2, P2X4/P2X7, and CX3CR1 receptors on microglia causes phosphorylation of p38 in microglia, leading to increased synthesis and release of TNF*α*, IL-1*β*, and BDNF.

The p38 MAPK pathway can be activated by a host of molecules known to increase pain sensitivity, including the proinflammatory cytokines TNF*α* and IL-1*β*, CCL2 (also known as monocyte chemoattractant protein 1 (MCP-1)), fractalkine (CX3CL1), inducible nitric oxide synthase (iNOS), and matrix metalloprotease-9 (MMP-9) as well as the ATP receptors P2X4 and P2X7 [40–47]. As shown in Figure 2, some of these microglial activators, such as ATP, CCL2, fractalkine, and MMP-9, could be released from primary afferent neurons [48, 49]. Once activated, the p38 pathway induces the expression of proinflammatory transcription factors, enzymes, and signaling molecules, including NFkappaB, COX2, iNOS, BDNF, TNF*α*, IL-1*β*, and IL-6 [38, 41, 50]. p38 activation in microglia also results in increased release of BDNF and TNF*α* in microglia [22]. Microglial production of proinflammatory cytokines can further recruit microglia, activate surrounding astrocytes, and promote the sensitization of central nervous system nociceptive circuits.

## 4. Dorsal Horn Microglial-Synapse Interactions in the Context of Persistent Pain

The development of central sensitization in persistent pain is characterized by increased excitatory synaptic transmission and decreased inhibitory synaptic transmission in the dorsal horn of the spinal cord. In order to modulate pain sensitivity and participate in central sensitization, glia must interact with neural pain circuits via modulation of neurotransmission. Glial mediators can modulate synaptic transmission at very low concentrations. While neurotransmitters such as glutamate, GABA, and glycine produce synaptic effects at *μ*M concentrations, glial cytokines, chemokines, and growth factors can affect synaptic activity at nM concentrations [51–53]. Accumulating evidence indicates a critical role of TNF*α*, IL-1*β*, and BDNF, all of which are released from activated microglia, in inducing the hyperactivity of dorsal horn neurons and thus in the development of pain hypersensitivity primarily in the setting of neuropathic pain, through modulation of both excitatory and inhibitory neurotransmission in the dorsal horn [40, 54, 55]. 

### 4.1. TNF*α*

TNF*α* is present both in healthy brain tissue and in disease states. TNF*α* is known to play a role in synaptic plasticity, which has been studied mainly in hippocampal slices. Glial TNF*α* has been shown to enhance synaptic efficacy by increasing the surface expression of GluR1-possessing AMPA receptors via TNFR1-mediated PI3 K activation [56, 57]. Glial TNF*α* also causes endocytosis of GABA_A_ receptors resulting in a decrease in inhibitory synaptic currents [57]. Homeostatic synaptic scaling of excitatory synapses increases their strength in response to network activity reduction or decreases their strength in response to increased network activity. In response to decreases in network activity, glial TNF*α* was shown to increase AMPA-mediated currents by increasing the number of calcium permeable AMPA receptors at the cell surface [58]. However, its role may be more permissive rather than instructive in this change [59]. 

**Figure 3:**
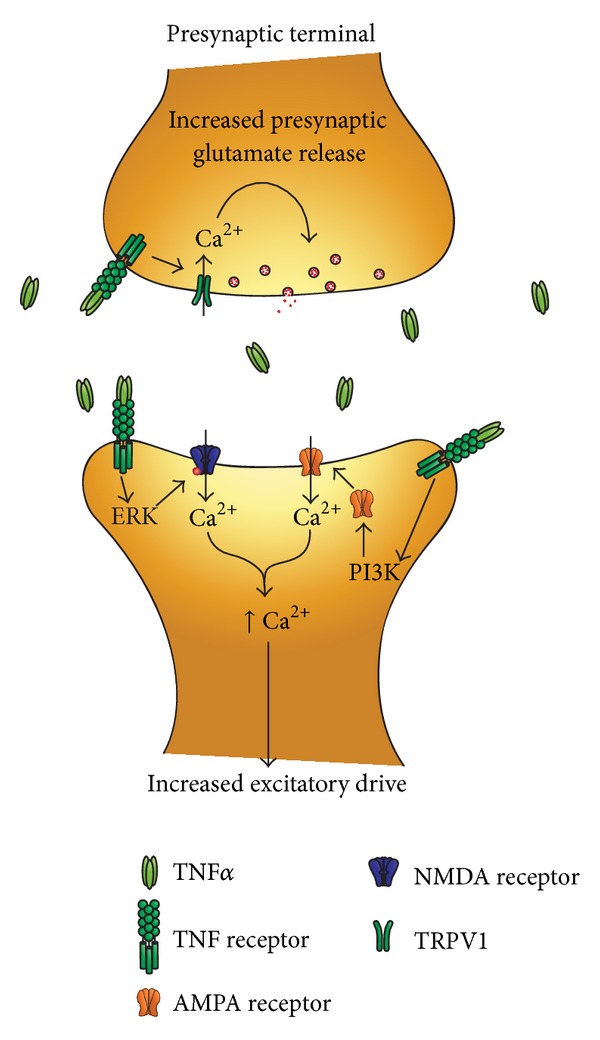
Schematic of TNF*α* induced potentiation of spinal cord synaptic transmission. Microglial release of TNF*α* increases excitatory neurotransmission in the dorsal horn via both presynaptic and postsynaptic mechanisms. At presynaptic sites, TNF*α* increases glutamate release via TRPV1 activation and there will be a subsequent increase in intracellular Ca^2+^. At postsynaptic sites, TNF*α* increases the activity of AMPA and NMDA receptors via activation of PI3 K and ERK on glutamatergic neurons to increase excitatory drive.

The effects of TNF*α* have also been studied in the dorsal horn of the spinal cord (see Figure 3). Intrathecal injection of TNF*α* causes the development of thermal and mechanical hyperalgesia [51]. To investigate the synaptic mechanisms of TNF*α*, many studies have used *ex vivo* spinal cord slice preparation. Incubation with TNF*α* increases the frequency of spontaneous excitatory postsynaptic currents (sEPSCs) in lamina II excitatory interneurons [60]. This could be indicative of a change in presynaptic glutamate release. TNF*α* increases sEPSC frequency via activation of transient receptor potential cation channel subfamily V member 1 (TRPV1) in presynaptic terminals, possibly through activation of adenylyl cyclase, protein kinase (PKA), or extracellular signal-related kinase (ERK) [60]. Activation of TRPV1 results in increased presynaptic calcium influx and, therefore, increased vesicular glutamate release [60].

TNF*α* also acts on the postsynaptic neurons in the spinal cord. In a carrageenan model of inflammation, TNF*α* recruited Ca^2+^ permeable AMPA receptors to dorsal horn neurons resulting in increased sEPSC amplitude [61]. NMDA currents in lamina II neurons are also enhanced by application of TNF*α* [51], and TNF*α* increases NMDA receptor (NMDAR) activity through phosphorylation of ERK in dorsal horn, neurons [62]. Thus via pre- and post synaptic mechanisms TNF*α* increases excitatory neurotransmission in the dorsal horn.

In spinal cord slices, TNF*α* not only enhances sEPSCs but also suppresses the frequency of spontaneous inhibitory postsynaptic currents (sIPSCs) [63]. This was found to be mediated by a decrease in spontaneous action potentials in GABAergic neurons via activation of TNF receptor 1 (TNFR1) and activation of p38 MAPK [63]. Neurons in the dorsal horn possess both TNFR1 and 2 (TNFR2), however, TNFR1 seems to make a greater contribution to enhancing nociceptive signaling in the dorsal horn [64]. 

In spinal cord slices from TNFR1 KO mice, TNF*α* was unable to elicit increases in sEPSCs or increases in NMDA currents [64]. However in TNFR2 KO mice, TNF*α* was still able to produce a small increase in sEPCS, and it elicited a normal increase in NMDA currents [64]. Both TNFR1 and TNFR2 knockout (KO) animals show decreased pain behavior in response to complete Freund's adjuvant and formalin induced inflammatory pain as well as intrathecal injection of TNF*α* [64]. Thus, microglial release of TNF*α* in the dorsal horn both enhances excitatory neuronal/synaptic activity and suppresses inhibitory neuronal/synaptic activity to enhance central sensitization primarily through the activation of TNFR1 on nociceptive dorsal horn neurons.

Long-term potentiation (LTP) in the spinal cord is implicated in pathological pain [65]. LTP in the spinal cord can be triggered by stimulation of the primary afferent fibers with the typical high-frequency titanic stimulation [66] and also by low-frequency stimulation (a more physiological firing pattern of nociceptors) [67] as well as by formalin or capsaicin administration to the paw or by nerve injury [16, 17, 67]. TNF*α* is also important for the induction of spinal LTP [68], and both TNFR1 and TNFR2 are required for titanic stimulation-induced LTP [60, 69]. While the prevailing view is that TNF*α* release from glia activates TNF receptors on neurons to promote LTP, new evidence has recently been found that TNF receptor expression on glial cells is also necessary for the generation of spinal LTP [69]. In the presence of fluorocitrate, a pharmacological blocker of glial activation, TNF*α* failed to potentiate AMPAR-mediated synaptic currents [69]. This suggests that there is an intermediate step which may facilitate TNF*α*-induced sensitization of dorsal horn neurons. TNF*α* could act on glial cells to elicit the release of additional glial mediators to facilitate its pronociceptive effects. Furthermore, pretreatment of slices with the microglial selective inhibitor minocycline inhibits LTP produced by high-frequency stimulation, however, application of TNF*α* reversed the effect [70], again suggesting that microglial TNF*α* release is important for long-term synaptic plasticity. 

It is important to note that the effects of TNF*α* vary among regions of the CNS particularly at high pathological concentrations. While constitutive TNF*α* release may be permissive for plastic changes in neurotransmission, the activation of microglia can result in 10-fold higher TNF*α* concentrations and, at these high concentrations (greater than 0.3 nM), TNF*α* may change its mode of action [71]. When cultured in the presence of microglia, astrocytic glutamate released is dramatically amplified [72]. In hippocampal slices, high concentrations of TNF*α* may cause prostaglandin E2 mediated glutamate release from astrocytes [73] to the degree that it causes excitotoxic damage in neurons [72]. It has been repeatedly demonstrated that at high concentrations (10–100 ng/mL), TNF*α* inhibited hippocampal LTP [74–76]. However, in spinal dorsal, horn both low and high concentrations of TNF*α* increased C-fiber induced LTP in nerve-injured rats [68].

### 4.2. IL-1*β*

IL-1*β* is induced in astrocytes, neurons, and microglia in inflammatory and neuropathic pain [77–80]. Intrathecal administration of IL-1*β* induces both thermal and mechanical hyperalgesia [81, 82]. Inhibition of IL-1*β* signaling through administration of IL-1 receptor antagonists decreased allodynia in neuropathic pain models [77, 83–85]. IL-1*β* enhances nociceptive activity in the dorsal horn through some of the same mechanisms as TNF*α*. Application of IL-1*β* to spinal cord slices increases sEPSC frequency, which indicates enhanced excitatory neurotransmission through increased release of neurotransmitter [51]. Importantly, IL-1*β*, but not TNF*α*, increased the amplitude of sEPSCs, suggesting additional postsynaptic regulation [51]. Also similar to TNF*α*, IL-1*β* enhances NMDA receptor currents in dorsal horn neurons [86], albeit by a different mechanism. IL-1*β* induces phosphorylation of the NR1 subunit of the NMDA receptor [87, 88]. NR1 is an essential subunit of the NMDA receptor, and phosphorylation of NR1 increases NMDA-mediated inward currents. In both inflammatory and neuropathic pain models, phosphorylation of NR1 increases NMDA receptor activity, facilitating excitatory neurotransmission in the dorsal horn [89–91].

IL-1*β* also suppresses inhibitory neurotransmission in the dorsal horn. Application of IL-1*β* to spinal cord slices inhibits both the frequency and the amplitude of spontaneous postsynaptic currents (sIPSCs) [51]. Therefore it functions via both pre- and postsynaptic mechanisms. Furthermore, application of IL-1*β* is capable of reducing both GABA and glycine mediated currents in dorsal horn neurons [51]. Overall, IL-1*β* enhances pronociceptive neurotransmission both through enhancing excitatory neurotransmission and suppressing inhibitory neurotransmission. 

### 4.3. BDNF

Brain derived neurotrophic factor (BDNF) is a secreted protein and part of the family of neurotrophins which act on neurons to promote survival, growth, and differentiation of new neurons and synapses [92]. In the brain, BDNF is important for synaptic plasticity and long-term memory [93, 94]. However, in the spinal cord following nerve injury, it plays a deleterious role. 

Following nerve injury, microglia upregulate their expression of the ionotropic ATP receptor P2X4 concurrently with the development of allodynia [95]. P2X4 receptor stimulation results in the activation of the p38 MAPK pathway and increases the synthesis and release of BDNF from microglial cells [96]. Application of BDNF to the spinal cord is capable of producing mechanical allodynia in otherwise naïve animals [97]. Furthermore, intrathecal administration of microglia activated by ATP into naïve animals produces mechanical allodynia, which can be alleviated by blockade of the BDNF receptor TrkB [95]. Thus microglial produce BDNF following nerve injury and BDNF is sufficient to produce pain behavior. 

Microglial derived BDNF contributes to pain hypersensitivity through the disinhibition of nociceptive processing in the dorsal horn. BDNF acts on lamina I pain transmission neurons; neurons that carry the output message of the dorsal horn to higher brain centers where pain is perceived. BDNF, through the activation of its receptor TrkB, decreases expression of the potassium-chloride cotransporter 2 (KCC2) resulting in a rise in intracellular chloride [53, 98]. This shift in the neuronal anion gradient is sufficient to suppress inhibition in the majority of lamina I projection neurons, and in some neurons the shift is large enough that their response to application with GABA becomes excitatory rather than inhibitory [53, 99, 100]. The outcome is suppression of the intrinsic inhibitory circuit in the dorsal horn. Protein levels of KCC2 are down-regulated in the spinal cord following both spinal cord injury and nerve-injury models in parallel with the development of thermal and mechanical hypersensitivity [101, 102].

In naïve animals, lamina I projection neurons respond to painful but not innocuous stimuli. The suppression of inhibitory drive and thus exaggerated responses of lamina I neurons to normally noxious stimuli explains the development of hyperalgesia, an exaggerated response to normally noxious stimuli, but it does not explain the development of allodynia, the painful response to normally innocuous stimuli. Persistent pain sufferers report three cardinal features of their pain: hyperalgesia, allodynia, and spontaneous pain. *In vivo* recordings from lamina I projection neurons show that following nerve-injury, these neurons begin to respond to nonnoxious stimuli, increase their response to noxious stimuli, and discharge spontaneously [103]. Though the mechanism is not fully elucidated, these results can be recapitulated by disrupting the chloride homeostasis of lamina I projection neurons as well as by the addition of ATP stimulated microglia, which presumably secrete BDNF [103]. Thus microglial derived BDNF may participate further in the development of persistent pain conditions, yet the entire mechanism remains to be elucidated. 

## 5. Concluding Remarks

Hyperactivity of peripheral nociceptive fibers due to inflammation or injury causes the release of factors such as CCL2, ATP, and fractalkine into the dorsal horn of the spinal cord [48, 49]. The binding of these factors to their respective receptors CCR2, P2X4, P2X7, and CX3CR1 on microglia causes activation of proinflammatory cascades and often gliosis [40–47]. p38 MAPK acts as a signal integrator of microglia stimulation. Activation of p38 results in the production and release of cytokines, chemokines, and proinflammatory mediators [22] that amplify pronociceptive signals in the dorsal horn.

We examined the mechanisms of three key signaling molecules released by microglial cells in the context of pain: TNF*α*, IL-1*β*, and BDNF. TNF*α* and IL-1*β* increase presynaptic release of excitatory neurotransmitter as well as increasing excitatory postsynaptic currents through the recruitment of AMPA receptors and enhancing NMDA currents [51, 60–62, 86]. Both also suppress inhibitory neurotransmission through distinct mechanisms. TNF*α* decreases spontaneous activity in GABAergic neurons while IL-1*β* suppresses GABA and glycine mediated inhibitory currents [51, 63]. BDNF in the dorsal horn functions a wholly different mechanism to suppress inhibitory neurotransmission. Microglial release of BDNF decreases expression of KCC2 [53, 98]. This shifts the chloride gradient of the cell sufficiently to make the normally inhibitory currents resulting from GABA application negligible or even excitatory. Therefore, these proinflammatory mediators promote central sensitization through multiple mechanisms to increase excitatory drive and decrease inhibitory drive in dorsal horn nociceptive circuitry.

TNF*α*, IL-1*β*, and BDNF also affect long-term neuronal plasticity in the dorsal horn. Activation of their respective receptors, TNFR1, IL-1, and trkB, on neurons leads to the phosphorylation of ERK which can enter the nucleus and produce phosphorylation of cAMP response element binding protein (CREB) [51, 104–106]. Activation of the transcription factor CREB occurs following nerve injury [107]. Its activation leads to the transcription of genes such as COX-2, NK-1, and trkB which increase neuronal excitability and further promote sensitization [106, 108, 109]. These proinflammatory mediators encourage both short-term and long-term sensory plasticity in the dorsal horn resulting in an increase in ascending nociceptive neurotransmission to the brain. This central sensitization is a key component in the development of hyperalgesia and allodynia associated with persistent pain disorders, and microglia are emerging as a key contributor to central sensitization. 

## 6. Future Directions

Recently, studies have found that microglia, like peripheral macrophages, are capable of distinct functional states referred to as classical or alternative activation. Classical activation is initiated by IFN*γ*, IL-1*β*, and LPS which increase production and release of proinflammatory molecules such as TNF*α* and IL-1*β* [110, 111]. Alternative activation is induced by IL-4, IL-13, IL-10, and TGF*β* which activate anti-inflammatory cascades or tissue repair mechanisms in microglia [110, 112]. The anti-inflammatory actions of IL-4 and IL-13 are mainly a result of their ability to antagonize changes induced by proinflammatory cytokines and suppress the production of IL-1*β* and TNF*α* from microglia [113–117]. Additionally, IL-4 treatment increases the mRNA expression of repair genes, which are also markers of alternative activation in macrophages, such as arginase 1and mannose receptor 1, found in inflammatory zone 1 and Ym1 [118, 119]. Administration of IL-10 and TGF*β* or uptake of apoptotic cells induces an alternative activation state sometimes called acquired deactivation, that as well as being anti-inflammatory is also an immunosuppressive state [110]. However, the majority of studies on alternative activation have been performed using peripheral macrophages, and anti-inflammatory signaling cascades in microglia have not been widely investigated and remain poorly understood. Furthermore, it also remains to be seen if these anti-inflammatory cytokines are capable of regulating synaptic plasticity in the dorsal horn. 

A second class of anti-inflammatory, proresolution mediators are the lipid derivatives resolvins and neuroprotectins. Administration of resolvin E1 (RvE1) or neuroprotectin/protectin D1 (NPD1/PD1) can block TNF*α* induced synaptic plasticity as well as long-term potentiation in the dorsal horn [120–122]. Additionally, RvE1 is capable of blocking TNF*α* induced increases of postsynaptic NMDA currents via regulation of ERK [121, 122]. RvE1 and NPD1 can further block LPS-induced TNF*α* release in microglia [123, 124]. While blocking proinflammatory cytokine production remains a major avenue of clinical pain management, anti-inflammatory and proresolving lipid mediators may provide new avenues for controlling clinical pain.
